# Supplementary material for: Circadian photoreceptor CRYPTOCHROME promotes wakefulness under short winter-like days via a GABAergic circuitry
Source: eLife. 2026 Oct 8;12:RP92608. doi: 10.7554/eLife.92608 (PMC13649261; doi:10.7554/eLife.92608)
Supplement: Peer review history

# Editor report: eLife Assessment

**DOI:** 10.7554/eLife.92608.4.sa0

**Authors:** Krishna Melnattur

**Affiliations:** Ashoka University India

**Keywords:** Solid, Valuable

Winter months with short days are commonly associated with seasonal depression and hypersomnolence; the mechanisms behind this hypersomnolence however, remain unclear. Chen and colleagues identify a genetic basis for this phenomenon in the fly Drosophila - mutations in the circadian photoreceptor cryptochrome resulted in increased sleep under short photoperiods. These findings are **valuable** insights into the genetic mechanisms regulating sleep under short days. There is **solid** evidence that cryptochrome acts in GABAergic neurons, but only limited evidence for the proposed site of action. Further work with better techniques will be needed to identify the precise site of action of cryptochrome.

# Referee report: Reviewer #1 (Public review)

**DOI:** 10.7554/eLife.92608.4.sa1

Summary:

In this paper, Chen et al. identified a role for the circadian photoreceptor CRYPTOCHROME (CRY) in promoting wakefulness under short photoperiods. This research is potentially important as hypersomnolence is often seen in patients suffering from SAD during winter times. The mechanisms underlying these sleep effects are poorly known.

Strengths:

The authors clearly demonstrated that mutations in cry lead to elevated sleep under 4:20 Light-Dark (LD) cycles. Furthermore, using RNAi, they identified GABAergic neurons as a primary site of CRY action to promote wakefulness under short photoperiods. They then provide genetic and pharmacological evidence demonstrating that CRY acts on GABAergic transmission to modulate sleep under such conditions.

Weaknesses:

The authors then went on to identify the neuronal location of this CRY action on sleep. This is where this reviewer is much more circumspect about the data provided. The authors hypothesize that the l-LNvs which are known to be arousal promoting may be involved in the phenotypes they are observing. To investigate this, they undertook several imaging and genetic experiments.

While the authors have made improvements in this resubmitted manuscript, there are still multiple concerns about the paper. I think the authors provide enough evidence suggesting that CRY plays a role in sleep under short photoperiod. The data also supports that CRY acts in GABAergic neurons. However, the identity of the GABAergic neurons involved in this CRY dependent sleep mechanism remains unclear. Similarly, whether l-LNvs are the target of this GABA mediated sleep regulation under short photoperiod is not fully demonstrated. The data presented suggests that but does not conclusively prove it.

# Referee report: Reviewer #3 (Public review)

**DOI:** 10.7554/eLife.92608.4.sa2

Summary:

In humans, short photoperiods are associated with hypersomnolence. The mechanisms underlying these effects is, however, unknown. Chen et al. use the fly Drosophila to determine the mechanisms regulating sleep under short photoperiods. They find that mutations in the circadian photoreceptor cryptochrome (cry) increase sleep specifically under short photoperiods (e.g. 4h light : 20 h dark). They go on to show that cry is required in GABAergic neurons and that the effects of the cry mutation on sleep are mediated by alterations in GABA signalling. Further, they suggest that the relevant subset of GABAergic neurons are the well-studied small ventral lateral neurons that they suggest inhibit the arousal promoting large ventral neurons via GABA signalling.

Strengths:

Genetic analysis to show that cryptochrome (but not other core clock genes) mediates the increase in sleep in short photoperiods, and circuit analysis to localise cry function to GABAergic neurons.

Weaknesses:

The authors' have substantially revised their manuscript, and the manuscript is much better for the revisions. However, the idea that the sLNvs are GABAergic is unfortunately still not well supported by the data. The authors have acknowledged the limitations of their methods though which is very welcome, and a substantial improvement.

# Referee report: Reviewer #4 (Public review)

**DOI:** 10.7554/eLife.92608.4.sa3

Summary:

Short photoperiod is an important experimental manipulation in neurobiology, endocrinology, and metabolism studies. However, the molecular mechanisms by which short photoperiod gives rise to behavioral phenotypes that are seen in seasonal affective disorders remain unknown. Using the classic circadian model organism Drosophila, this study examines short photoperiod-induced hypersomnolence and identifies the circadian photoreceptor cryptochrome as a regulator of GABAergic tone within the clock neural circuit to promote wakefulness under short photoperiod conditions. The discovery has broad implications for understanding how short photoperiod modulates neural inhibition in circadian circuits in regulating sleep.

Strengths:

The Drosophila model provided a powerful platform to dissect the molecular mechanisms underlying short photoperiod-induced hypersomnolence. A battery of behavioral, imaging, circuit-manipulation approaches were employed to test the novel hypothesis that the circadian photoreceptor cryptochrome modulates GABAergic tone within the clock neural circuit to promote wakefulness under short photoperiod conditions.

Weaknesses:

The current model proposed by the authors suggests that the small ventral lateral neurons of the Drosophila clock circuit are GABAergic; however, this remains unclear. At present, the field lacks sufficient data and validated reagents to definitively establish the GABAergic identity of these neuropeptidergic neurons.

# Author comment: Author response

**DOI:** 10.7554/eLife.92608.4.sa4

**Authors:** Lixia Chen, Danya Tian, Chang Su, Luoying Zhang

**Affiliations:** Huazhong University of Science and Technology Wuhan China; Huazhong University of Science and Technology Wuhan China; Huazhong University of Science and Technology Wuhan China; Huazhong University of Science and Technology Wuhan China

The following is the authors’ response to the previous reviews

**Public Reviews:**

**Reviewer #1 (Public review):**

Summary:

In this paper, Chen et al. identified a role for the circadian photoreceptor CRYPTOCHROME (CRY) in promoting wakefulness under short photoperiods. This research is potentially important as hypersomnolence is often seen in patients suffering from SAD during winter times. The mechanisms underlying these sleep effects are poorly known.

Strengths:

The authors clearly demonstrated that mutations in cry lead to elevated sleep under 4:20 Light-Dark (LD) cycles. Furthermore, using RNAi, they identified GABAergic neurons as a primary site of CRY action to promote wakefulness under short photoperiods. They then provide genetic and pharmacological evidence demonstrating that CRY acts on GABAergic transmission to modulate sleep under such conditions.

Weaknesses:

The authors then went on to identify the neuronal location of this CRY action on sleep. This is where this reviewer is much more circumspect about the data provided. The authors hypothesize that the l-LNvs which are known to be arousal promoting may be involved in the phenotypes they are observing. To investigate this, they undertook several imaging and genetic experiments.

While the authors have made improvements in this resubmitted manuscript, there are still multiple concerns about the paper. I think the authors provide enough evidence suggesting that CRY plays a role in sleep under short photoperiod. The data also supports that CRY acts in GABAergic neurons. However, there are still major issues with the quality of the confocal images presented throughout the paper. In many cases it appears that the images are oversaturated with poor resolution, making it hard to understand what is going on. In addition, none of the drivers used in this study are specific to the neurons the authors aim to manipulate. Therefore, the identity of the GABAergic neurons involved in this CRY dependent sleep mechanism remains unclear. Similarly, whether l-LNvs are the target of this GABA mediated sleep regulation under short photoperiod is not fully demonstrated. The data presented suggests that but does not prove it.

Major concerns:

(1) While the authors provided sleep parameters like consolidation or waking activity for some experiments. These measurements are still not shown for several experiments (for example Figures 2E, 3, 4, 5, and 6). These data are essential, these metrics must be reported for all sleep experiments.

These metrics have now been added to Fig.2 S4 and 5, Fig.3 S1-3, Fig.4 S2 and 3, Fig.5 S2 and 4, as well as Fig.6 S1.

(2) Line 144 "We fed flies with agonists of GABA-A (THIP) and GABA-B receptor (SKF-97541) (Ki and Lim, 2019; Matsuda et al., 1996; Mezler et al., 2001). Both drugs enhance sleep in WT," The proper citation is needed here, Dissel et al., 2015 PMID:25913403. Both THIP and SKF-97541 were used in that paper.

Thank you for pointing this out. We have modified our manuscript accordingly.

(3) Figure 2C and 2F: it appears that the control data is the same in both panels. That is not acceptable.

Thank you for pointing this out. We are now using data from control flies that were monitored in the same experiments as the experimental groups.

(4) Figure 4A: With the quality of the images, it is impossible to assess whether GABA levels are increased at the l-LNvs soma.

We apologize for the poor quality. Unfortunately, the GABA immunostaining does not work very well in our hands and thus the background is high. We have now commented on this issue in the fourth paragraph of discussion and have toned down our conclusions regarding the GABAergic s-LNv—l-LNv circuitry in this revised version of the manuscript.

(5) Fig 4 S1A shows colabeling of l-LNvs and Gad1-Gal4 expressing neurons. They are almost 100% overlapping signals. This would indicate that the l-LNvs are GABAergic themselves, or that there is a problem with this experiment.

Fig 4 S1A demonstrates the expression pattern of SYT-GFP driven by Gad1GAL4, which should label the synaptic terminals of GABAergic neurons. Therefore, the labeling observed at l-LNvs suggest that GABAergic neurons project to l-LNvs. This is further validated by the GRASP and trans-Tango experiments.

(6) Fig 4 S1B: Again, I can see colabelling of the GFP and PDF staining, suggesting that Gad1-Gal4 expresses in l-LNvs.

Fig 4 S1B demonstrates anatomical sites where GABAergic neurons project to and form synaptic connections with PDF neurons. Therefore, GFP signals at the l-LNvs suggest that these cells receive synaptic inputs from GABAergic neurons, echoing the results shown in Fig 4 S1A.

(7) Line 184: "Consistently, knocking down Rdl in the l-LNvs rescues the long sleep phenotype of cry mutants (Figure 4-figure supplement 1D)." This statement is incorrect as the driver used for this experiment, 78G01-GAL4 is not specific to the l-LNvs, so it is possible that the phenotypes observed are not coming from these neurons.

Thank you for pointing this out. We have modified our manuscript to note this.

(8) Figure 4G-K: None of these manipulations are specific to the l-LNvs. The authors describe 10H10-GAL4 and 78G01-GAL4 as l-LNvs specific tools, but this is not the case. Why not use the SS00681 Split-GAL4 line described in Liang et al., 2017 PMID: 28552314? It is possible that some of the effects reported in this manuscript are not caused by manipulating the l-LNvs.

Thank you for pointing this out. We have now modified our manuscript to avoid misleading remarks. We have used SS00681 Split-GAL4 to express TrpA1 but did not observe any substantial effect on sleep duration under short photoperiod. Therefore, we did not use this line for further experiments.

(9) Similarly for the manipulation of s-LNvs, the authors cannot rule out effect that are coming from other cells as R6-GAL4 is not specific to s-LNvs.

We have now modified our manuscript to avoid misleading remarks.

(10) The staining presented in Fig 5 S1 is not very convincing. Difficult to see whether Gad1-GAL4 only expresses in the s-LNvs.

We have now quantified the GFP signal in the l-LNvs and s-LNVs in Fig.5 S1B and D. As can be seen, the s-LNvs show prominent signal above the background while the l-LNvs do not.

**Reviewer #3 (Public review):**

Summary:

In humans, short photoperiods are associated with hypersomnolence. The mechanisms underlying these effects is however, unknown. Chen et al. use the fly Drosophila to determine the mechanisms regulating sleep under short photoperiods. They find that mutations in the circadian photoreceptor cryptochrome (cry) increase sleep specifically under short photoperiods (e.g. 4h light: 20 h dark). They go on to show that cry is required in GABAergic neurons and that the effects of the cry mutation on sleep are mediated by alterations in GABA signalling. Further, they suggest that the relevant subset of GABAergic neurons are the well-studied small ventral lateral neurons that they suggest inhibit the arousal promoting large ventral neurons via GABA signaling

Strengths:

Genetic analysis to show that cryptochrome (but not other core clock genes) mediates the increase in sleep in short photoperiods, and circuit analysis to localise cry function to GABAergic neurons.

Weaknesses:

The authors' have substantially revised their manuscript, and the manuscript is better for the revisions. However, the conclusion that the sLNvs are GABAergic is unfortunately still not well supported by the data. A key sticking point remains the anti GABA immunostaining, and specific driver lines for sLNvs and lLNvs.

The authors should tone down their conclusions to reflect the fact that their data, as presented, does not support the model that cry acts in sLNvs to modulate GABA signalling onto lLNvs and thus modulate sleep.

Thank you for the comments. We have now toned down our conclusions regarding the GABAergic s-LNv—l-LNv circuitry in this revised version of the manuscript in the Introduction, Results and Discussion.

**Reviewer #4 (Public review):**

Summary:

Short photoperiod is an important experimental manipulation in neurobiology, endocrinology, and metabolism studies. However, the molecular mechanisms by which short photoperiod gives rise to behavioral phenotypes that are seen in seasonal affective disorders remain unknown. Using the classic circadian model organism Drosophila, this study examines short photoperiod-induced hypersomnolence and identifies the circadian photoreceptor cryptochrome as a regulator of GABAergic tone within the clock neural circuit to promote wakefulness under short photoperiod conditions. The discovery has broad implications for understanding how short photoperiod modulates neural inhibition in circadian circuits in regulating sleep.

Strengths:

The Drosophila model provided a powerful platform to dissect the molecular mechanisms underlying short photoperiod-induced hypersomnolence. A battery of behavioral, imaging, circuit-manipulation approaches was employed to test the novel hypothesis that the circadian photoreceptor cryptochrome modulates GABAergic tone within the clock neural circuit to promote wakefulness under short photoperiod conditions.

Weaknesses:

The current model proposed by the authors suggests that the small ventral lateral neurons of the Drosophila clock circuit are GABAergic; however, this remains unclear. At present, the field lacks sufficient data and validated reagents to definitively establish the GABAergic identity of these neuropeptidergic neurons.

Thank you for the comments. We have now toned down our conclusions regarding the GABAergic s-LNv—l-LNv circuitry in this revised version of the manuscript.

**Recommendations for the authors:**

The manuscript has improved after revisions. However, the evidence in support of the claim that the sLNVs secrete GABA onto the lLNvs remains unconvincing. The evidence that loss of cry in GABAergic neurons modulates sleep is solid. However, the authors' claim that the sLNVs are the relevant GABAergic neurons is not sufficiently backed up by the evidence presented. We suggest that the authors tone down their conclusions to reflect this.

Thank you for the comments. We have now toned down our conclusions regarding the GABAergic s-LNv—l-LNv circuitry in this revised version of the manuscript in the Introduction, Results and Discussion.

**Reviewer #3 (Recommendations for the authors):**

Minor points:

(1) The authors suggest that the effects of cry on sleep and mediated by the Rdl receptor, and use the GABA agonist THIP as support of this argument. However THIP acts on the Lcch3 and Grd receptors, not Rdl

Thank you for pointing this out. We have modified relevant discussion accordingly.

(2) In several instances (e.g. line 66, line 148), the authors use 'consistently' in the sense of 'consistent with previous data'. It would be better if they rephrase this.

This has been fixed.

**Reviewer #4 (Recommendations for the authors):**

It is my pleasure to serve as a reviewer for this revised manuscript. The authors have carefully revised the manuscript in response to the critiques raised by all previous reviewers and have used all the available reagents to conduct additional experiments to assess the GABAergic properties of the small ventral lateral neurons (sLNv). Although it remains unclear in the field whether sLNvs co-transmit GABA, this study raises this possibility within an interesting biological relevant context. I recommend this manuscript for final publication.

Thank you for your comments.
